# On Max-Semistable Laws and Extremes for Dynamical Systems

**DOI:** 10.3390/e23091192

**Published:** 2021-09-09

**Authors:** Mark P. Holland, Alef E. Sterk

**Affiliations:** 1 College of Engineering, Mathematics and Physical Sciences, Harrison Building, Streatham Campus, University of Exeter, North Park Road, Exeter EX4 4QF, UK; 2 Bernoulli Institute for Mathematics, Computer Science and Artificial Intelligence, University of Groningen, P.O. Box 407, 9700 AK Groningen, The Netherlands; a.e.sterk@rug.nl

**Keywords:** extreme value theory, max-semistable laws, tail index, extremal index, dynamical systems, 37D99, 60F99

## Abstract

Suppose (f,X,μ) is a measure preserving dynamical system and ϕ:X→R a measurable observable. Let $X_{i}=\phi \circ f^{i-1}$ denote the time series of observations on the system, and consider the maxima process $M_{n}:=\max\left\{X_{1},\ldots ,X_{n}\right\}$. Under linear scaling of $M_{n}$, its asymptotic statistics are usually captured by a three-parameter generalised extreme value distribution. This assumes certain regularity conditions on the measure density and the observable. We explore an alternative parametric distribution that can be used to model the extreme behaviour when the observables (or measure density) lack certain regular variation assumptions. The relevant distribution we study arises naturally as the limit for max-semistable processes. For piecewise uniformly expanding dynamical systems, we show that a max-semistable limit holds for the (linear) scaled maxima process.

## 1. Introduction

### 1.1. Overview on the Theory of Extremes

Consider a stationary stochastic process $\left(X_{n}\right)$ on a probability space (Ω,P,F), where Ω is the sample space and P is a probability measure on the sigma-algebra F. Study of the maxima process $M_{n}=\max_{k\;\leq \;n}X_{k}$ is the topic of *Extreme Value Theory* (EVT), and has wide applications, e.g., in weather, climate and financial modelling [1,2]. Within EVT, a particular problem is concerned with understanding the limiting behaviour of the process $M_{n}$ as n→∞, either in distribution, or almost surely. This has relevance to statistical modelling applications and prediction of extremes [3]. In this article, we consider distributional convergence of $M_{n}$, and consider the possible limit distributions governing the rescaled process $a_{n}\left(M_{n}-b_{n}\right)$, for real-valued sequences $a_{n}$, and $b_{n}$. This is a natural problem to consider, and is in direct analogy to establishing (for example) the Central Limit Theorem property for normalised sums of random variables. In particular, we seek the existence of sequences $a_{n},b_{n}\in R$ such that

(1) $$P\left\{a_{n}\left(M_{n}-b_{n}\right)\leq u\right\}\to G\left(u\right),$$

for some non-degenerate distribution function G(u), −∞<u<∞.

For independent, identically distributed (i.i.d.) processes $\left(X_{n}\right)$, the limit law *G* (when it exists) is known to take three forms: Fréchet, Weibull and Gumbel [1,2,3]. Up to scale and location changes, they can be summarised through the *generalised extreme value* (GEV) distribution $G_{\xi }\left(u\right)$ defined as follows:

(2) $$G_{\xi }\left(u\right)=\left\{\begin{array}{cc} \exp\left\{-\;{\left(1+\xi u\right)}^{-\frac{1}{\xi }}\right\} & \mathrm{if}\;\xi \neq 0,\\ \exp\left\{-\;e^{-u}\right\} & \mathrm{if}\;\xi =0. \end{array}\right.$$

The parameter ξ∈R is referred to as the *tail* or *shape* parameter, and is of key interest in statistical estimation and fitting of the GEV distribution. The Gumbel distribution corresponds to ξ=0, Fréchet to ξ>0, and Weibull to ξ<0. For a given probability distribution $F_{X}\left(u\right):=P\left(X\leq u\right)$, the existence of a limit $G_{\xi }\left(u\right)$ depends on the asymptotic regular variation properties of $F_{X}$, or in particular the ‘tail’ ${\bar{F}}_{X}\left(u\right):=1-F_{X}\left(u\right)$ as $u\to u_{F}\leq \infty$. Here, $u_{F}=\sup\left\{v\in R:F_{X}\left(v\right)<1\right\}$. For example, suppose $u_{F}=\infty$, and there exists β>0 such that for all ℓ>0,

(3) $$\lim_{u\to \infty }\frac{{\bar{F}}_{X}\left(\ell u\right)}{{\bar{F}}_{X}\left(u\right)}=\ell^{-\beta }.$$

If we put $a_{n}=F^{-1}\left(1-1/n\right)$ and $b_{n}=0$, then the limit for $P\left(a_{n}\left(M_{n}-b_{n}\right)<u\right)\to G_{\xi }\left(u\right)$ can be shown to exist, with $G_{\xi }\left(u\right)=e^{-u^{-\xi }}$ (of GEV type) and ξ=1/β. Thus, any probability distribution function satisfying Equation (3) belongs to the domain of attraction of a Fréchet law with tail parameter ξ=1/β. Formulation of general conditions on $F_{X}\left(u\right)$ and existence/construction of the norming sequences $a_{n}$ and $b_{n}$ to permit convergence of (normalised) maxima to a GEV distribution are discussed in [1]. However, there are wide classes of distributions for which there are *no* normalising sequences to permit convergence in distribution of $a_{n}\left(M_{n}-b_{n}\right)$. A particular class we introduce are the max-semistable distributions.

### 1.2. Max-Semistable Laws and Corresponding Evt

Here, we introduce the class of max-semistable distributions. Given a random variable *X* with distribution function $F_{X}$, we say that *X* is in the domain of (partial) attraction to a max-semistable distribution function G(u) if there exists a strictly increasing sub-sequence $k_{n}$, such that $k_{n+1}/k_{n}\to c\geq 1$, and normalising constants $a_{n}$, $b_{n}$ with

$$F_{X}{\left(\frac{u}{a_{n}}+b_{n}\right)}^{k_{n}}\to G\left(u\right).$$

The distribution function G(u), when it exists, is characterised by the equivalent property: there exists c>1, γ>0 and β∈R with

$$G\left(u\right)=G{\left(\frac{u}{\gamma }+\beta \right)}^{c}.$$

If convergence takes place along the full sequence $k_{n}=n$ (so that c=1), then we refer to *G* as max-stable. In particular, the distribution functions represented by the classical GEV distribution in Equation (2) are max-stable. A representative of a max-semistable distribution $G\left(u\right):=G_{\xi ,\nu }\left(u\right)$ takes the following functional form:

(4) $$G_{\xi ,\nu }\left(u\right)=\left\{\begin{array}{cc} \exp\left\{-\;{\left(1+\xi u\right)}^{-\frac{1}{\xi }}\nu \left(\log{\left(1+\xi u\right)}^{-\frac{1}{\xi }}\right)\right\} & \mathrm{if}\;1+\xi u>0,\;\xi \neq 0,\\ \exp\left\{-\;e^{-u}\nu \left(u\right)\right\} & \mathrm{if}\;u\in (-\infty ,\infty ),\;\xi =0, \end{array}\right.$$

where ν is a positive, bounded and periodic function with period $c_{\nu }=\log c>0$. When ν≡1, then $G_{\xi ,\nu }\left(u\right)$ takes the previous form of a (max-stable) GEV distribution described by Equation (2). The max-semistable distributions capture the limit laws for linear scaling sequences of $M_{n}$, especially when the probability distribution function (or measure) governing $X_{n}$ has oscillation behaviour in the tails. Indeed, if $M_{n}$ is the maximum for an i.i.d. sequence $\left(X_{n}\right)$, then for the sequences $a_{n}$, $b_{n}$ and $k_{n}$ above, we have

$$P\left(a_{n}\left(M_{k_{n}}-b_{n}\right)\leq u\right)\to G\left(u\right),$$

for all values of *u* that are continuity points of *G*.

In the i.i.d. case, the domain of attraction for a particular $G_{\xi ,\nu }$ is understood in terms of regularity of the tails for $F_{X}\left(u\right)$ as $u\to u_{F}$; see [4,5]. For example, in the case ξ>0, the distribution function $F_{X}\left(u\right)$ will be in the domain of attraction for $G_{\xi ,\nu }$ if the following holds: there exists a function $\tilde{F}\left(u\right)$ regularly varying with index α=−1/ξ, sequences $a_{n}$, $b_{n}$, and $x_{*}\in R$ a continuity point of ν such that $\Theta \left(u\right):={\bar{F}}_{X}\left(u\right)/\tilde{F}\left(u\right)$ satisfies

$$\lim_{n\to \infty }\frac{\Theta (a_{n}u+b_{n})}{\Theta (a_{n}x_{*}+b_{n})}=\frac{\nu (\log x)}{\nu (\log x_{*})}.$$

Moreover, the corresponding sequence $k_{n}$, with $k_{n+1}/k_{n}\to c$, can be made explicit:

$$k_{n}=\left\lfloor \frac{\nu \left(\log x_{*}\right){\left(x_{*}\right)}^{-\alpha }}{{\bar{F}}_{X}\left(a_{n}x_{*}+b_{n}\right)}\right\rfloor .$$

**Example** **1.** *Consider the distribution*

$$F_{X}\left(u\right)=1-u^{-\alpha }\left(1+\epsilon \sin\left(\frac{2\pi }{c}\log u\right)\right)\;\mathrm{with}\;\epsilon <\frac{c\alpha }{2\pi +c\alpha }.$$

*If we put $a_{n}=e^{cn/\alpha }$, $b_{n}=0$, $x_{*}=1$ and $\tilde{F}\left(u\right)=u^{-\alpha }$, then*

$$\lim_{n\to \infty }\frac{\Theta (e^{cn}x)}{\Theta (e^{c})}=1+\epsilon \sin\left(\frac{2\pi }{c}\log u\right),$$

* and so $\nu \left(u\right)=1+\epsilon \sin\left(\frac{2\pi }{c}\log u\right)$. We now have to consider $k_{n}$. We have*

(5) $$\begin{array}{cc} P(M_{k_{n}}<u/a_{n}+b_{n}) & ={\left(1-{\left(e^{\frac{cn}{\alpha }}u\right)}^{-\alpha }\left(1+\epsilon \sin\left(\frac{2\pi }{c}\log\left(e^{cn}u\right)\right)\right)\right)}^{k_{n}}\\ \; & ={\left(1-e^{-cn}u^{-\alpha }\left(1+\epsilon \sin\left(\frac{2\pi }{c}\log u\right)\right)\right)}^{k_{n}}\\ \; & =\exp\left\{k_{n}e^{-cn}u^{-\alpha }\nu \left(u\right)\right\}+O\left(k_{n}e^{-2cn}\right). \end{array}$$

*Choosing $k_{n}=\left\lfloor e^{cn}\right\rfloor$, we obtain*

$$P\left(M_{k_{n}}<u/a_{n}+b_{n}\right)\to \exp\left\{u^{-\alpha }\nu \left(u\right)\right\}.$$

*Thus, this example is in the domain of attraction of $G_{\xi ,\nu }\left(u\right)$, with ξ=1/α and $\nu \left(u\right)=1+\epsilon \sin\left(\frac{2\pi }{c}\log u\right)$ (Notice that relative to earlier notation, the period of ν is precisely $c_{\nu }=c$). Clearly, $k_{n}$ satisfies the regularity condition $k_{n+1}/k_{n}\to e^{c}$.*

The tail of the distribution ${\bar{F}}_{X}\left(u\right)$ satisfies

$$\underset{u\to \infty }{lim sup}u^{\alpha }{\bar{F}}_{X}\left(u\right)=1+\epsilon ,\;\underset{u\to \infty }{lim inf}u^{\alpha }{\bar{F}}_{X}\left(u\right)=1-\epsilon ,$$

and admits infinite oscillation over log-periodic windows. In particular, the function $u^{\alpha }{\bar{F}}_{X}\left(u\right)$ is log-periodic with period $e^{c}$. (Recall that a function M:R→R is log-periodic with period γ>0 if M(γx)=M(x) for all x∈R.)

**Example** **2.** *Consider the distribution function with tail ${\bar{F}}_{X}\left(u\right)=\exp\left\{-e^{-u-\epsilon \sin u}\right\}$ for some 0<ϵ<1. Then it can be shown that this function is in the domain of attraction of $G_{\xi ,\nu }\left(u\right)$, with ξ=0; see [4].*

However, the oscillation property of the distribution function within the domain of attraction can be subtle as the next example illustrates.

**Example** **3.** *Consider the distribution function with tail*

$${\bar{F}}_{X}\left(u\right)=u^{-\alpha }\ell \left(u\right)\;with\;\ell \left(u\right)=\exp\left\{\sqrt{\log u}\sin\left(\sqrt{\log u}\right)\right\},\;\left(u\to \infty \right).$$

*Then ${\bar{F}}_{X}\left(u\right)$ is regularly varying with index −α. Thus, this distribution is in the domain of attraction of a max-stable GEV distribution with limit representation $G_{\xi }\left(u\right)=e^{-u^{-\alpha }}$. Note, however, that the function ℓ(u) is both slowly varying, and satisfies infinite oscillation in the sense that*

$$\underset{u\to \infty }{lim sup}\ell \left(u\right)=\infty ,\;\underset{u\to \infty }{lim inf}\ell \left(u\right)=0.$$

We remark further that if a distribution function has slowly varying tails, such as ${\bar{F}}_{X}\left(u\right)={\left(\log u\right)}^{-\beta }$ with β>0, then $F_{X}\left(u\right)$ is not in the domain of attraction of a max-stable, nor a max-semistable law [1,4].

The remainder of this paper is organised as follows. In Section 2, we state our main results. This includes the statement of Theorem 1 on existence of a max-semistable law for piecewise uniformly expanding dynamical systems. We show that the limit law obtained depends on the regularity of the observables on the system, and on the regularity of the invariant density. In Section 2.4, we discuss the role of the extremal index. This is a further parameter that captures certain clustering behaviour [1,3], and is not applicable to the i.i.d. case. The extremal index is not directly incorporated in the GEV representation, and its computation requires analysis of the dependency structure of the process. In Section 3, we analyse the performance of statistical estimation schemes, such as the L-moments method for estimating the parameters of the limiting max-semistable GEV distribution. We also compute the extremal index and compare to theoretical results.

## 2. Convergence to a Max-Semistable Law for Dynamical Systems

We now consider a measure preserving dynamical system f:X→X, on the probability space (X,μ,F). Here, X⊂R, F a Borel σ-algebra on X, and μ is an *f*-invariant probability measure supported on X. Given an observable ϕ:X→R, i.e., a measurable function, we consider the stationary stochastic process $X_{1},X_{2},\cdots$ defined as

(6) $$X_{i}=\phi \circ f^{i-1},\;i\geq 1,$$

and its associated maximum process $M_{n}$ defined as

(7) $$M_{n}=\max\left\{X_{1},\cdots ,X_{n}\right\}.$$

As in the i.i.d. case, much attention has been to determine the existence of sequences $a_{n},b_{n}\in R$ such that

(8) $$\nu \left\{x\in X:a_{n}\left(M_{n}-b_{n}\right)\leq u\right\}\to G\left(u\right),$$

for some non-degenerate distribution function G(u), −∞<u<∞. Under general assumptions on the observable function, the measure density and the mixing properties of the dynamical system, it is found that the sequences $a_{n}$, $b_{n}$ and limit *G* are determined in much a similar way as to the i.i.d. case.

Here, the distribution function tail ${\bar{F}}_{X}\left(u\right)$ takes the form ${\bar{F}}_{f}\left(u\right):=\mu \left\{\phi \left(x\right)>u\right\}$. The regularity of ${\bar{F}}_{f}\left(u\right)$ depends on the regularity of the measure μ, and on the regularity of the observable ϕ. We focus on one-dimensional dynamical systems, and consider those with an absolutely continuous invariant measure μ. For μ-a.e. x∈X the density ρ(x) is well defined and takes values in (0,∞). There may be exceptional points where $\rho \left(\tilde{x}\right)\in \left\{0,\infty \right\}$, or is undefined. For the observable function ϕ:X→R, we consider those which are maximised at a distinguished point $\tilde{x}\in X$. Moreover, we consider observable functions of the form $\phi \left(x\right)=\psi (\mathrm{dist}\left(x,\tilde{x}\right))$, where dist(·,·) denotes the Euclidean distance on X and ψ:[0,∞)→R is a monotone decreasing function. Functions of this form have been the main focus in the study of extremes for one-dimensional dynamical systems; see [6]. For example, it can be shown that the max-stable GEV limit distributions are applicable for describing the statistics of extremes in the cases: (i) ψ(u)=−logu; (ii) $\psi \left(u\right)=u^{-\alpha }$, and (iii) $\psi \left(u\right)=C-u^{\alpha }$, (with α>0). The problem we consider is the case where ${\bar{F}}_{f}\left(u\right)$ is not regularly varying, and hence not in the domain of attraction of a classical max-stable GEV distribution. For one-dimensional dynamical systems where the density of μ is a smooth function (e.g., the density is μ-a.e. Hölder continuous), the regularity of ${\bar{F}}_{f}\left(u\right)$ (or lack thereof) depends on the regularity of the observable function ϕ (through ψ). Hence, we seek conditions on the dynamical system process, and observable function ψ for which a max-semistable law limit exists. We cannot use the same methods of proof as in the i.i.d. case, since the dynamical system processes are dependent.

Going beyond one-dimensional dynamical systems, proving existence (or otherwise) of a max-stable GEV distribution limit is non-trivial. This is a relevant problem to consider, especially from a practical viewpoint of using dynamical systems for weather and climate models. For non-uniformly hyperbolic systems, e.g., those giving rise to chaotic attractors as in [7,8], the regularity considerations of the invariant measure will feature prominently in the determination of the limit law for the extremes (if such a limit law exists). Numerical results indicate slow or oscillatory convergence in the estimation of the tail parameter; see [6,9,10,11]. Within these references, it is shown that lack of regular variation for the function ${\bar{F}}_{f}\left(u\right)$ is possible. This remains the case even if the observable function is sufficiently smooth, in the sense of $\phi \left(x\right)=\psi (\mathrm{dist}\left(x,\tilde{x}\right))$, and the function ψ regularly varying. The lack of regular variation of ${\bar{F}}_{f}\left(u\right)$ is due to the fractal, and (approximate) self-similar structure of the chaotic attractor. In particular, the invariant measure μ is longer absolutely continuous with respect to volume (Lebesgue) measure. Hence, it is natural to ask the validity of a max-semistable GEV distribution limit description for the extremes. We discuss this further in Section 4.

### 2.1. Main Results

Suppose that f:X→X is a piecewise expanding map, with finitely many pieces of continuity. For simplicity, we take X=[0,1]. We assume that there is a partition $P=\{I_{1},\ldots ,I_{m}\}$ such that *f* is differentiable on each $I_{k}$, k≤m. Let $P_{n}$ be the corresponding partition for $f^{n}$. We distinguish between finite and countable partitions. In the case of a finite partition P, there is a $\delta_{0}>0$ such that every partition element of P has a diameter of at least $\delta_{0}$. In the case where the partition P is countable, we assume that there is a $\delta_{0}>0$ such that for all *n* holds $|f^{n}\left(I\right)|\geq \delta_{0}$ whenever $I\in P_{n}$.

We assume that *f* is uniformly expanding, i.e., that there is a constant λ>1 such that $|f^{\prime }|\geq \lambda$. Moreover, we assume that *f* has bounded distortion, and that μ is an ergodic measure μ with exponential decay of correlations for functions of bounded variation against $L^{1}$. This means that there exists a constant C>0 such that

$$x,y\in I\in P_{n}\;\Rightarrow \;C^{-1}\leq \frac{Df^{n}\left(x\right)}{Df^{n}\left(y\right)}\leq C$$

and for functions $\varphi_{1},\varphi_{2}:X\to R$

$$\left|\int \varphi_{1}\cdot \varphi_{2}\circ f^{j}\;d\mu -\int \varphi_{1}\;d\mu \int \varphi_{2}\;d\mu \right|\leq C\tau_{1}^{-j}{∥\varphi_{1}∥}_{\mathrm{BV}}{∥\varphi_{2}∥}_{1}$$

for some $\tau_{1}>1$. Here, the density of the measure μ should be a function of bounded variation (BV) and ${∥\cdot ∥}_{\mathrm{BV}}$ denotes the BV-norm [12]. Recall that the $L^{1}$-norm is defined as ${∥\varphi ∥}_{1}=\int_{X}\left|\varphi \right|\;d\mu$.

Examples of systems satisfying our assumption are piecewise expanding maps with finitely many pieces and an absolutely continuous invariant measure μ, such as the β-transformation x↦βxmod1, (β>1); the Gauss map x↦1/xmod1; or the first return map to $\left[\frac{1}{2},1\right)$ for a Manneville–Pomeau map [13] with an absolutely continuous invariant measure μ. For more details about the statistical properties of these maps see [12,14]. We consider specific case studies in Section 3. We state the following result.

**Theorem** **1.** *Suppose that f:X→X is a piecewise uniformly expanding interval map, with ergodic measure μ. Given $\tilde{x}\in X$, suppose that $\phi \left(x\right)=\psi (\mathrm{dist}\left(x,\tilde{x}\right))$, with ψ:[0,∞)→R monotone decreasing. Suppose that there exists $\tilde{F}\left(u\right)$, regularly varying with index −α, a periodic function ν and sequences $a_{n}$, $b_{n}$ such that*

$$\lim_{n\to \infty }\frac{\Theta (a_{n}u+b_{n})}{\Theta (a_{n}x_{*}+b_{n})}=\frac{\nu (\log x)}{\nu (\log x_{*})},$$

* where $\Theta \left(u\right):={\bar{F}}_{f}\left(u\right)/\tilde{F}\left(u\right)$, and $x_{*}$ a continuity point of ν. Then for μ-a.e. $\tilde{x}\in X$, there exists a sequence $k_{n}$ with $k_{n+1}/k_{n}\to e^{c}\geq 1$ (where c is the period of ν), and*

$$\mu \left\{x\in X:a_{n}\left(M_{k_{n}}\left(x\right)-b_{n}\right)\leq u\right\}\to \exp\left\{-u^{-\alpha }\nu \left(\log u\right)\right\}.$$

We make several remarks on Theorem 1; it is proved in Section 2.2. The first remark is that an example function $F_{f}\left(u\right)$ that fits the hypothesis of Theorem 1 is given by

$${\bar{F}}_{f}\left(u\right)=u^{-\alpha }\left(1+\epsilon \sin\left(\frac{2\pi }{c}\log u\right)\right)\;\mathrm{with}\;\epsilon <\frac{\alpha c}{2\pi +\alpha c}.$$

It is straightforward to generalise to other functional forms. Another example includes:

$${\bar{F}}_{f}\left(u\right)=e^{-\gamma \beta^{-\lfloor \log x\rfloor }},\;\gamma ,\beta >0,$$

which is connected to the St. Petersburg distribution; see [5]. In a dynamical system setting, this type of limit distribution arises in the context of hitting time statistics to cylinder sets; see [15]. Note that the observable ϕ is defined implicitly through the function $F_{f}\left(u\right)=\mu \left\{\phi \left(x\right)>u\right\}$. In general it is not possible to give an explicit formula for ϕ (or ψ) even when the density of μ is explicit. The problem is inverting $F_{f}\left(u\right)$. If $\phi \left(x\right)=\psi (\mathrm{dist}\left(x,\tilde{x}\right))$ is made explicit, such as specifying $\psi \left(u\right)=u^{-\alpha }M\left(\log u\right)$ for some periodic function M(u), then the problem is to determine the regularity $F_{f}$. This becomes relevant for dynamical systems, where it is natural to specify ϕ first (rather than $F_{f}$). We state the following corollary.

**Corollary** **1.** *Suppose that f:X→X is a piecewise uniformly expanding interval map, with ergodic measure μ. Given $\tilde{x}\in X$, suppose that $\phi \left(x\right)=\psi (\mathrm{dist}\left(x,\tilde{x}\right))$, where ψ:[0,∞)→R and satisfies $\psi \left(u\right)=u^{-\alpha }M\left(\log u\right)$. The function M is assumed periodic with period c, and differentiable with $M^{\prime }\left(\log u\right)<\alpha M\left(\log u\right)$. Then for μ-a.e. $\tilde{x}\in X$, there exists a sequence $k_{n}$ with $k_{n+1}/k_{n}\to e^{c}\geq 1$, and*

$$\mu \left\{x\in X:e^{-c\alpha n}M_{k_{n}}\left(x\right)\leq u\right\}\to \exp\left\{-2\rho \left(\tilde{x}\right)u^{-\frac{1}{\alpha }}M_{0}\left(\log u\right)\right\},$$

* where $M_{0}\left(u\right)$ also has period c, and $\rho (\tilde{x})$ is the density of μ at $\tilde{x}$.*

The corollary is proved in Section 2.3. To keep the exposition concise, we have focused on piecewise uniformly expanding (interval) maps. It is possible to generalise to dynamical systems which are not uniformly expanding, such as the dynamical systems considered in [16,17,18]. The main purpose of our results is to demonstrate that max-semistable laws are the natural limits to consider for the maxima process, especially for observables that lack regular variation properties. The results we obtain are commensurate with the i.i.d. case. See also [19] for results in the context of certain stationary processes, building upon [20,21].

For hyperbolic systems, such as those considered in [7,8], we make further remarks in Section 4. In the context of semistable laws for suitably normalised Birkhoff sums (rather than extremes); see recent work of [22,23].

### 2.2. Proof of Theorem 1

The proof of Theorem 1 uses the blocking method adapted from [16,21]. See also ([6] Chapter 6), in particular Proposition 6.3.3 within. We summarise the approach as follows. Given *n*, consider integers p,q,t defined so that n∼q(p+t) as n→∞. We take $p=q∼\sqrt{n}$ and $t={\left(\log n\right)}^{2}$, but other rates are possible. We now divide up our process in blocks of size *p*, and take *q* such blocks. Each consecutive block will be separated by a time scale *t*. Block i≤q consists of the time series $\left\{X_{j-1+i(p+t)}\right\}$ for j=1,…p. Using the fact that the process is stationary, and an application of the inclusion-exclusion principle, the maxima of each block satisfies:

(9) $$1-p\mu \left(X_{1}>u_{n}\right)\leq \mu \left(M_{p}\leq u\right)\leq 1-p\mu \left(X_{1}>u_{n}\right)+\sum_{i=1}^{p}\sum_{j\neq i,j=1}^{p}\mu \left(X_{j}\geq u,X_{i}\geq u\right).$$

Since *t* represents a correlation time-lag it is natural to replicate the i.i.d. argument leading to an estimate of the form:

$$\left|\mu \left(M_{n}\leq u_{n}\right)-{\left(1-p\mu \left(X_{1}>u_{n}\right)\right)}^{q}\right|\leq E\left(p,q,t\right),$$

where the error term E(p,q,t) is composed of three significant terms, which we write as

$$E\left(p,q,t\right)=E_{1}+E_{2}+E_{3}.$$

- An error term $E_{1}$ which depends on the decay of correlations associated to separating the blocks by lag *t*. This is bounded by
  $$E_{1}\leq C\left(p,q\right)∥\varphi_{1}{∥}_{BV}{∥\varphi_{2}∥}_{L^{1}}\tau_{1}^{-n}$$
  where C(p,q) is power law in *n* when $p=q∼\sqrt{n}$ and $\tau_{1}>1$ is the exponential decay of correlation decay rate. The functions $\varphi_{1}=\varphi_{2}$ are indicator functions of the set $\{X_{1}>u_{n}\},$ and have $L^{\infty }$-norm of 1, bounded variation norm of 2. Hence, $E_{1}\to 0$ exponentially fast as n→∞
- An error term $E_{2}$ associated to the decomposition in (9). This is bounded as follows
  $$E_{2}\leq n\sum_{j=2}^{p}\mu \left(X_{1}>u_{n},X_{j}>u_{n}\right).$$
  For observables of the form $\phi \left(x\right)=\psi (\mathrm{dist}\left(x,\tilde{x}\right))$, it is shown that for μ-a.e. $\tilde{x}\in X$ that $E_{2}=O\left(n^{-\gamma_{1}}\right)$ for some $\gamma_{1}>0$. See [18].
- A remainder error term of the form $\max\left\{p,qt\right\}\mu \left(X_{1}>u_{n}\right)$ which arises from the requirement that p,q,t are integers. By choice of p,q,t and $u_{n}$, we see that $E_{3}=O\left(n^{-\gamma_{2}}\right)$ for some $\gamma_{2}>0$.

Hence, there exists $\tilde{\gamma }>0$ such that

$${\left(1-p\mu \left(X_{1}>u/a_{n}+b_{n}\right)\right)}^{q}=\exp\left\{-n\mu \left(X_{1}>u/a_{n}+b_{n}\right)\right\}+O\left(n^{-\tilde{\gamma }}\right),$$

and therefore

$$\mu \left(M_{n}\leq u/a_{n}+b_{n}\right)={\left(1-p\mu \left(X_{1}>u/a_{n}+b_{n}\right)\right)}^{q}+O\left(n^{-\tilde{\gamma }}\right).$$

To complete the proof, we must relabel the sequence indexing. We choose $a_{n}$, $b_{n}$ so that

$$\lim_{n\to \infty }\frac{\Theta (a_{n}u+b_{n})}{\Theta (a_{n}x_{*}+b_{n})}=\frac{\nu (\log x)}{\nu (\log x_{*})},$$

and for $M_{n}$, we consider instead $M_{k_{n}}$. This means we take $p=q∼\sqrt{k_{n}}$. We obtain

(10) $$\mu \left(M_{n_{k}}\leq u/a_{n}+b_{n}\right)=\exp\left\{-k_{n}{\bar{F}}_{f}\left(u/a_{n}+b_{b}\right)\right\}+O\left(k_{n}^{-\gamma_{0}}\right),$$

for some $\gamma_{0}>0$. By choice of $a_{n}$, and since ν(logx) is a log-periodic function of log-period $e^{c}$, we can choose $k_{n}$ proportional to $e^{c}$ as required. This concludes the proof.

### 2.3. Proof of Corollary 1

To prove the corollary, it suffices to analyse the regularity of $\psi^{-1}\left(u\right)$, such as its periodicity and regular variation properties. The following lemma is elementary and sets up an equivalence for log-periodicity of regular varying functions and their inverses.

**Lemma** **1.** *Suppose that $\psi \left(u\right)=u^{-\alpha }M\left(\log u\right)$, where M is periodic with period c. Suppose that M is differentiable and $M^{\prime }\left(\log u\right)<\alpha M\left(\log u\right)$. Then $\psi^{-1}\left(u\right)$ admits the representation $\psi^{-1}\left(u\right)=u^{-1/\alpha }M^{♯}\left(\log u\right)$, where $M^{♯}\left(\log u\right)$ is also periodic with period c.*

The requirement $M^{\prime }\left(\log u\right)<\alpha M\left(\log u\right)$ ensures that ψ(u) is a monotone decreasing function, and is therefore injective so that $\psi^{-1}\left(u\right)$ is well defined. To show the periodicity property of $M^{♯}\left(u\right)$, we proceed as follows. First note that $\psi \left(e^{c}u\right)=e^{c\alpha }\psi \left(u\right)$, since $M\left(\log\left(e^{c}u\right)\right)=M\left(\log u\right)$. We now compare $\psi^{-1}\left(e^{c\alpha }x\right)$ with $\psi^{-1}\left(u\right)$:

$$\begin{array}{cc} \psi^{-1}\left(c^{\alpha }u\right) & =\{v:\;\psi \left(v\right)=e^{c\alpha }u\},\\ \; & =\{v:\;v^{\alpha }M\left(\log v\right)=e^{c\alpha }u\},\\ \; & =\{v:\;{\left(ve^{-c}\right)}^{\alpha }M\left(\log\left(e^{-c}v\right)\right)=u\},\\ \; & =e^{c}\psi^{-1}\left(u\right). \end{array}$$

Hence, $\psi^{-1}\left(u\right)=e^{\frac{c}{\alpha }}\psi^{-1}\left(u\right)$. Put $\psi^{-1}\left(u\right)=u^{\frac{1}{\alpha }}M^{♯}\left(\log u\right)$, for some real-valued function $M^{♯}\left(u\right)$. Then we see that $M^{♯}\left(\log\left(e^{c}u\right)\right)=M^{♯}\left(\log u\right)$ as required. This completes the proof.

### 2.4. On the Role of the Extremal Index

For dependent processes, a further important parameter of statistical relevance is the extremal index θ. It is defined as follows:

**Definition** **1.** *Suppose τ>0, and let $u_{n}\left(\tau \right)$ be a sequence such that*

(11) $$n\mu \{X_{1}>u_{n}\left(\tau \right)\}\to \tau ,\;n\to \infty .$$

*Then we say that an extreme value law with extremal index θ∈(0,1] holds for $M_{n}$ if*

(12) $$\mu \left\{M_{n}\leq u_{n}\left(\tau \right)\right\}\to e^{-\theta \tau },\;n\to \infty .$$

If $\left(X_{n}\right)$ is an i.i.d. process, then Equation (12) holds for θ=1. For dynamical systems, natural examples where the extremal index is non-trivial are for observables $\phi \left(x\right)=\psi (\mathrm{dist}\left(x,\tilde{x}\right))$ maximised at periodic points. Following, e.g., [15,24], versions of Theorem 1 can be shown to hold. To see where the extremal index arises more explicitly, consider the following example. Take $Y_{n}=\max\left\{X_{n},X_{n+1}\right\}$, where $\left(X_{n}\right)$ is an i.i.d. sequence with distribution function ${\bar{F}}_{X}\left(u\right)=u^{-1}M\left(\log u\right)$. We assume M is differentiable, periodic with period *c* and $M^{\prime }\left(\log u\right)<M\left(\log u\right)$. Defining $\Theta \left(u\right)=u{\bar{F}}_{X}\left(u\right)$, we get identically Θ(u)=M(logu). Thus, along the sequence $a_{n}=e^{cn}$, we have $\lim_{n\to \infty }\theta \left(e^{cn}u\right)=M\left(\log u\right)$. (We can take $x_{*}=1$.) If $M^{Z}$ denotes the maximum of a general random variable sequence $\left(Z_{n}\right)$, then we see that $M_{n}^{Y}=M_{n+1}^{X}$. Hence, taking $a_{n}=e^{cn}$ and $b_{n}=0$, we have

$$P\left(M_{k_{n}}^{Y}\leq u/a_{n}+b_{n}\right)=P\left(M_{k_{n}+1}^{X}\leq u/a_{n}+b_{n}\right)={\left(1-{\left(e^{cn}u\right)}^{-1}M\left(\log u\right)\right)}^{k_{n}+1}.$$

Now the convergence criteria to a max-semistable law are characterised by sequences $k_{n}$ satisfying the asymptotic ratio condition $k_{n+1}/k_{n}\to c^{\prime }$ for some $c^{\prime }\geq 1$. We can take $k_{n}=e^{nc}-1$. The limit distribution is represented by $G_{\xi ,\nu }$ with ξ=1 and ν=M(logu). Notice that this construction does not pick up the extremal index. This is due to the fact that the sequence $k_{n}$ can be defined up to arbitrary multiplication constants. In the max-stable case, we work precisely along the given sequence $k_{n}\equiv n$, and $a_{n}$, $b_{n}$ are chosen by the requirement $n{\bar{F}}_{X}\left(u/a_{n}+b_{n}\right)\to \tau$. If instead we took M(logu)≡1, then we would take $a_{n}=n$, $b_{n}=0$, and obtain

$$P\left(M_{k_{n}}^{Y}\leq u/a_{n}+b_{n}\right)\to e^{-\tau /2},$$

thus picking up an extremal index of 1/2.

From a practical viewpoint, the extremal index measures ‘clustering phenomena’ and this is a separate phenomenon associated to irregularity of the tails. We explore in the next section whether numerical methods still pick up the non-trivial extremal index, despite the extremal index itself not featuring directly in the limiting max-semistable GEV representation. We note that even in the classical max-stable GEV representation the extremal index is not formally incorporated. It is hidden within the scale and location parameters. Regarding Equation (12), the sequence $u_{n}\left(\tau \right)$ appearing within is not required to satisfy any particular regularity condition, i.e., as associated to a linear scaling distributional limit for $M_{n}$ (which indeed will not always exist).

## 3. Numerical Studies

In this section, we undertake simulation studies for dynamical system case studies, where the observable function is in the domain of attraction of a max-semistable GEV distribution. We estimate (numerically) the tail parameter, the extremal index, and discuss to what extent we can determine the periodicity of the function ν in the max-semistable GEV representation. The examples we consider are: i.i.d. random variables; uniformly expanding maps fitting the scope of Theorem 1 and observable functions within the scope of Corollary 1; certain non-uniformly expanding maps such as the logistic map and cusp map.

**Example** **4.** *Consider the distribution function*

$$F\left(u\right)=1-u^{-\alpha }\left(1+\epsilon \sin\left(\frac{2\pi }{c}\log u\right)\right),$$

* with α=4, c=1, and ϵ=0.35. We draw samples from this distribution via the time series $X_{i}=F^{-1}\left(U_{i}\right)$ where the $U_{i}$ are i.i.d. random variables with a uniform distribution on the interval [0,1]. The function $F^{-1}$ is computed numerically by solving the equation $F\left(X_{i}\right)=U_{i}$ using Newton’s method.*

*First, $10^{3}$ block maxima are extracted from a time series $\left(X_{i}\right)$ where the length of the blocks is allowed to vary. Next, the tail index ξ is estimated by the L-moments method [25]. In addition, an estimate for the 95% confidence interval is obtained by repeating the computations 50 times with different realizations. See [11,26] for further details. The extremal index θ is estimated by applying the the intervals estimator introduced in [27] to a time series of length $10^{4}$.*

**Figure 1:**
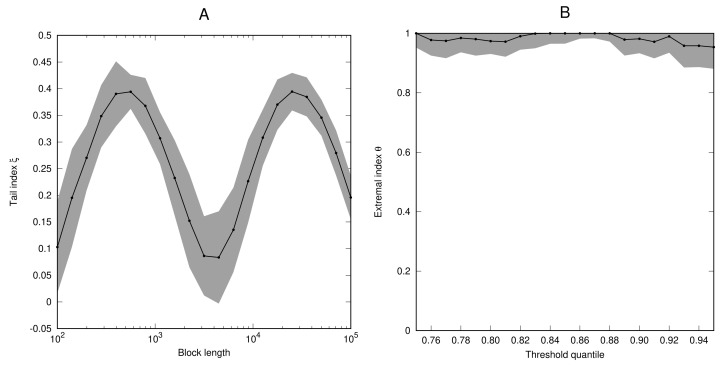
Numerical estimates of the tail index ξ (**A**) and the extremal index θ (**B**) for the process $\left(X_{i}\right)$ defined in Example 4. Grey bands mark the 95% confidence intervals around the obtained estimates.

*Figure 1 shows estimates for the tail index ξ as a function of the block length (panel A) and the extremal index θ as a function of the threshold quantile (panel B). The tail index strongly oscillates around the value $\xi =\frac{1}{4}$ when the block length is increased. The value ξ=1/4 is precisely the tail parameter in the max-semistable GEV distribution. However, the estimation scheme does not easily pick out the period of oscillation $c_{\nu }$ for the function ν. The estimated extremal index is close to 1 which is expected since the time series $\left(X_{n}\right)$ is an i.i.d. process. Also note that the estimates of θ are not very sensitive to the choice of the quantile threshold.*

**Figure 2:**
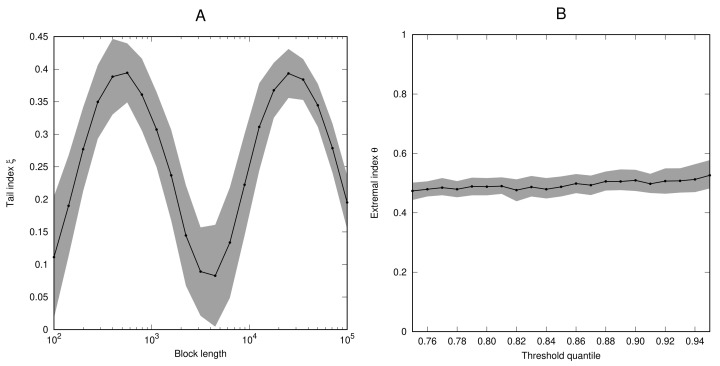
Numerical estimates of the tail index ξ (**A**) and the extremal index θ (**B**) for the process for the process $\left(Y_{i}\right)$ defined in Example 5.

**Example** **5.** *Next, we consider the process $\left(Y_{i}\right)$ given by $Y_{i}=\max\left\{X_{i},X_{i+1}\right\}$, where $\left(X_{i}\right)$ is the sequence from Example 4. Figure 2 again shows that the estimates of the tail index ξ as a function of the block length behave in a very similar way to Example 4. However, in this case, the process $\left(Y_{i}\right)$ is no longer i.i.d. and estimates for the extremal index are close to $\theta =\frac{1}{2}$.*

**Example** **6.** *In this example, we consider the proces $\left(X_{i}\right)$ defined in Equation (6) using the map f(x)=3xmod1 on the interval [0,1) and the observable $\phi \left(x\right)=\psi (\mathrm{dist}\left(x,\tilde{x}\right))$, where*

(13) $$\psi \left(u\right)=u^{-\alpha }M\left(u\right)\;\mathrm{and}\;M\left(u\right)=1+\epsilon \sin\left(\frac{2\pi }{c}\log u\right).$$

**Figure 3:**
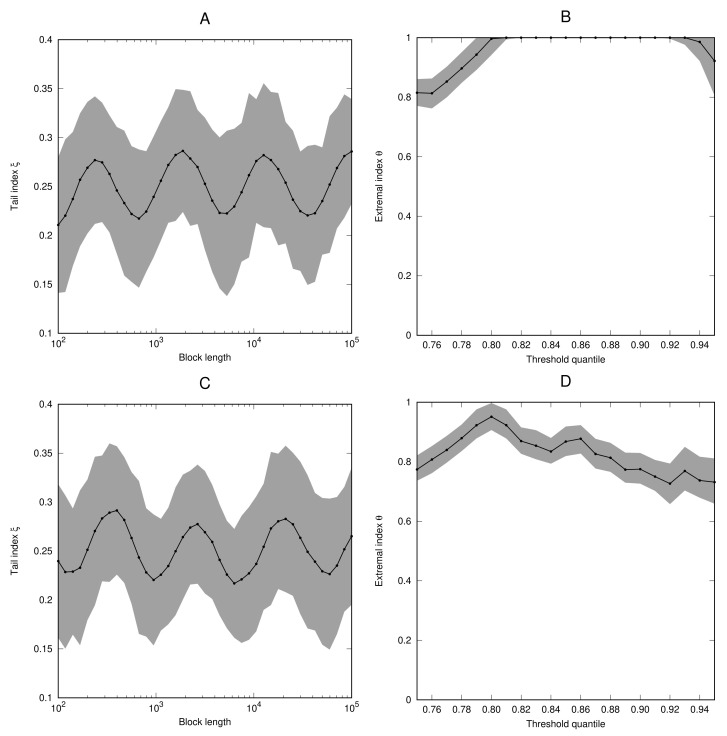
As Figure 1, but for the process $\left(X_{i}\right)$ defined in Example 6 with $\tilde{x}=\frac{1}{2}\sqrt{3}$ (**A**,**B**) and $\tilde{x}=\frac{1}{2}$ (**C**,**D**).

*For the parameter values α=0.25, ϵ=0.05, and c=2, the condition of Lemma 1 is satisfied. Figure 3 shows the estimates for the tail index and extremal index for the cases $\tilde{x}=\frac{1}{2}\sqrt{3}$ (which is a non-periodic point of f) and $\tilde{x}=\frac{1}{2}$ (which is a fixed point of f). In both cases, the estimates for the tail index oscillate around the value $\xi =\frac{1}{4}$ when the block length is increased. In the case $\tilde{x}=\frac{1}{2}\sqrt{3}$ the extremal index is very close to 1. In the case $\tilde{x}=\frac{1}{2}$, we have θ≈0.73, which compares well to the theoretically expected value which is given by*

$$\theta =1-\frac{1}{|f^{\prime }\left(\tilde{x}\right)|}=\frac{2}{3},$$

* see [24].*

**Figure 4:**
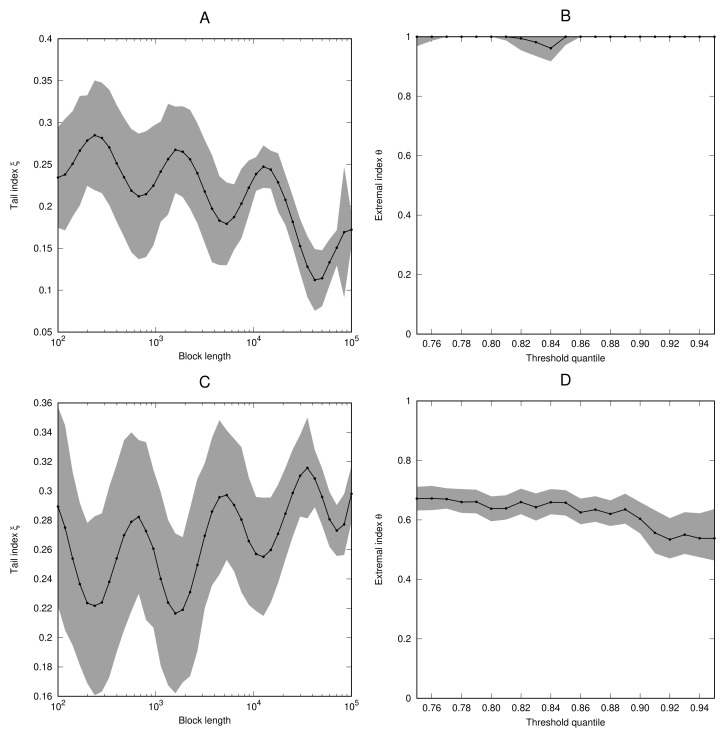
As Figure 1, but for the process $\left(X_{i}\right)$ defined in Example 7 with $\tilde{x}=\frac{1}{2}\sqrt{3}$ (**A**,**B**) and $\tilde{x}=\frac{3}{4}$ (**C**,**D**).

**Example** **7.** *As a more interesting example, we consider the process $\left(X_{i}\right)$ defined in Equation (6) using the logistic map f(x)=4x(1−x) on the interval [0,1]. We take the observable $\phi \left(x\right)=\psi (\mathrm{dist}\left(x,\tilde{x}\right))$, where ψ is defined in Equation (13), with the same parameter values as in Example 6. Figure 4 shows the estimates for the tail index and extremal index for the cases $\tilde{x}=\frac{1}{2}\sqrt{3}$ (which is a non-periodic point of f) and $\tilde{x}=\frac{3}{4}$ (which is a fixed point of f). In both cases, the estimates for the tail index oscillate when the block length is increased. However, contrary to Example 6, the oscillations do not occur around a particular value but an upward (resp. downward) trend can be observed. A possible explanation for this phenomenon might be that it takes longer for the oscillations to settle because of the fact that f is non-uniformly expanding. Although the density of the invariant measure, given by $\rho \left(x\right)=\frac{1}{\pi }{\left(x\left(1-x\right)\right)}^{-1/2}$, is a smooth function, it is the log-periodic oscillation in the observable function (via ψ) in Equation (13) that gives rise to the oscillations in the tail estimation. Corollary 1 applies to this example. In the case $\tilde{x}=\frac{1}{2}\sqrt{3}$ the extremal index is very close to 1. In the case $\tilde{x}=\frac{3}{4}$, we have θ≈0.53, which compares well to the theoretically expected value which is given by*

$$\theta =1-\frac{1}{|f^{\prime }\left(\tilde{x}\right)|}=\frac{1}{2},$$

* see [24].*

**Figure 5:**
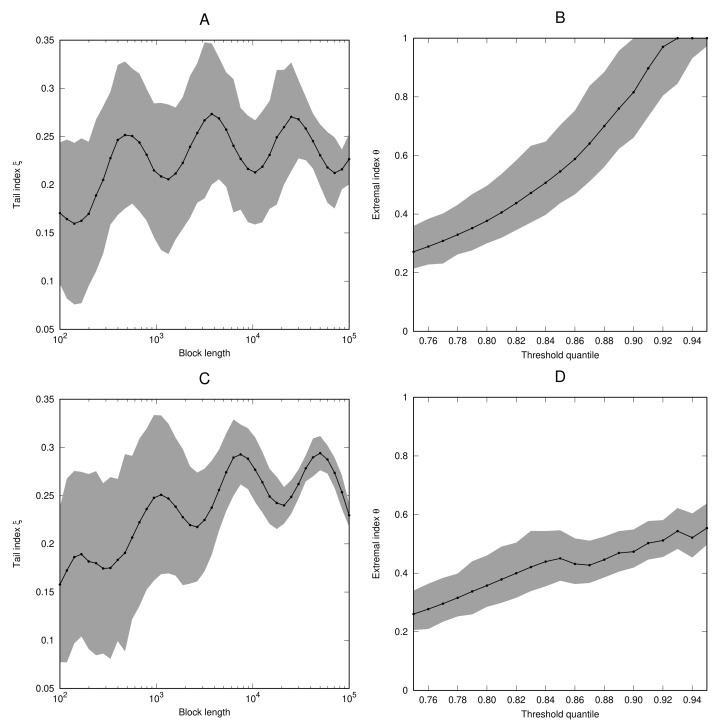
As Figure 1, but for the process $\left(X_{i}\right)$ defined in Example 8 with $\tilde{x}=\frac{1}{2}\sqrt{3}$ (**A**,**B**) and $\tilde{x}=3-\sqrt{8}$ (**C**,**D**).

**Example** **8.** *Finally, we consider the process $\left(X_{i}\right)$ defined by Equation (6) using the cusp map $f\left(x\right)=1-2\sqrt{\left|x\right|}$ on the interval [−1,1] and the observable $\phi \left(x\right)=\psi (\mathrm{dist}\left(x,\tilde{x}\right))$, where ψ is defined in Equation (13) and the same parameter values as in Example 6 are taken. Figure 5 shows the estimates for the tail index and extremal index for the cases $\tilde{x}=\frac{1}{2}\sqrt{3}$ (which is a non-periodic point of f) and $\tilde{x}=3-\sqrt{8}$ (which is a fixed point of f). In both cases, the estimates for the tail index oscillate when the block length is increased. As in Example 7 the oscillations also show upward and downward trends. In the case $\tilde{x}=\frac{1}{2}\sqrt{3}$ the extremal index is very close to 1, but as opposed to all the previous the extremal index depends rather sensitively on the chosen threshold quantile. A possible explanation for this phenomenon could be the intermittent nature of the map f; iterates visit neigbourhoods of the point x=−1 much more frequently than neighbourhoods of points x>0. In the case $\tilde{x}=3-\sqrt{8}$, we have θ≈0.55 when the threshold quantile is 0.95. This estimate compares well to the theoretically expected value which is given by*

$$\theta =1-\frac{1}{|f^{\prime }\left(\tilde{x}\right)|}=1-\sqrt{3-\sqrt{8}}\approx 0.59,$$

* see [24].*

## 4. Discussion

In this article, we have shown the existence of max-semistable limit laws for certain dynamical systems. For the systems we have considered, the existence on the type of limit law for the maxima process depends on the regularity of the observable function. For more general non-uniformly expanding (interval) maps, such as those that preserve an absolutely continuous invariant measure, then we expect similar conclusions to apply relative to Theorem 1 and Corollary 1. The corresponding results obtained would essentially depend on the regularity of the observable ϕ and the measure density in the vicinity of the maxima $\tilde{x}\in X$. As mentioned in Section 2, for dynamical systems giving rise to chaotic attractors, regularity considerations of the invariant measure will be important in determining the existence (or otherwise) of a limit law for the extremes, whether that limit law be max-stable, or max-semistable. Unless the fractal structure of the chaotic attractor is strictly self-similar, then establishing existence of a max-semistable law would depend on finer (statistical) self-similar properties of the attractor, and local properties of the invariant measure in the vicinity of the point $\tilde{x}$. This is the case when taking an observable function of the form $\phi \left(x\right)=\psi (\mathrm{dist}\left(x,\tilde{x}\right))$. See [6,9,10,11]. When a max-semistable law description is valid, an ongoing work is to explore statistical methods to capture more formally the periodic behaviour, such as the computation of the periodicity constant for ν. In the case of estimating the periodicity constant for i.i.d. processes; see [4].

In our studies, the numerical computation of the extremal index has conformed accurately to the theoretical results. As we have pointed out in Section 2.4, the extremal index does not appear (naturally) in the GEV representation, and therefore the oscillation behaviour of the periodic function ν within is unlikely to affect the computation of the extremal index. Numerical accuracy in extremal index estimation has been due to dynamical considerations, such as presence of a neutral fixed point discussed in Example 8.
